# Diagnostic Value of Copeptin in Patients with Suspected Pulmonary Embolism in Emergency Departments

**Published:** 2019-03

**Authors:** Alireza Abootalebi Ghahnavieh, Keihan Golshani, Mohammadsaleh Jafarpisheh, Milad Moaiednia, Mohammad Ali Memarzade, Asieh Maghami-Mehr

**Affiliations:** 1 Emergency Medicine Research Center, Al-Zahra Research Institute, Isfahan University of Medical Sciences, Hezarjarib Ave, Isfahan, Iran,; 2 Department of Statistics, Payam Noor University, Shiraz, Iran.

**Keywords:** Copeptin, Pulmonary Embolism, D-dimer, Validity

## Abstract

**Background:**

Pulmonary thromboembolism (PTE) is a serious and life-threatening condition. Diagnosis of PTE can be challenging in emergency departments, as there is no absolutely reliable biomarker for the diagnosis of PTE. Copeptin (COP) is a new biomarker, which may be valuable in the diagnosis of PTE; however, its role has not been well studied. In this study, we aimed to investigate the diagnostic value of COP in the diagnosis of PTE.

**Materials and Methods:**

This study was carried out on 102 patients suspected of PTE. The serum levels of D-dimer and COP were measured, and diagnosis of PTE was confirmed by CT angiography. Next, the prognostic value of D-dimer and COP was examined.

**Results:**

The area under the curve (AUC) of D-dimer was 0.581 with a standard error (SE) of 0.07 (P=0.34). Estimation of the validity of D-dimer showed that it is a highly sensitive (100%), but poorly specific (15.8%) test. Evaluation of the predictive value of this test showed that it has a positive predictive value of 20% and a negative predictive value of 100%. The AUC of COP was 0.423 with SE of 0.1 (P=0.44). Measurement of the validity of COP test showed that it is a poorly sensitive (50%) and specific (22.9%) test.

**Conclusion:**

COP is a new cardiovascular biomarker. However, the present findings did not confirm the prognostic value of this biomarker for the diagnosis of PTE.

## INTRODUCTION

Pulmonary thromboembolism (PTE) is an important clinical disease, which may be life-threatening, especially if it is not diagnosed or treated properly (1). PTE is a common emergency condition that can affect a large number of people (2). It presents with a wide range of clinical manifestations, ranging from chest pain and hypoxia to severe cardiovascular collapse (3). These clinical manifestations are non-specific, which make the early diagnosis of PTE difficult in many cases (2). On the other hand, undiagnosed PTE is a condition that can be extremely life-threatening, as a large number of PTE-related mortalities occur in the first hours of hospital admission (4).

Today, diagnosis of PTE is mainly based on radiological and laboratory studies (2). Computed tomography pulmonary angiography (CTPA) and ventilation-perfusion scan (VQ scan) are two common modalities, which are widely used to diagnose PTE. However, they have major limitations in the diagnosis of PTE. Although CTPA is a valuable diagnostic test, which can reliably detect or rule out PTE, the negative predictive value (NPV) of CTPA ranges from 60% to 96% (5, 6). Despite the high predictive value of CTPA and VQ scan, additional laboratory studies are necessary for confirming the diagnosis of PTE (6, 7).

There is no reliable biomarker for the diagnosis of PTE. Today, D-dimer is used for the diagnosis of PTE. Nevertheless, the positive predictive value (PPV) of D-dimer is low, which makes it a diagnostic test with low accuracy for the diagnosis of PTE (5, 7). Despite the availability of advanced technologies and new diagnostic tools, many patients with PTE remain undiagnosed or untreated worldwide (1, 5). Therefore, new diagnostic biomarkers are needed to promote rapid and accurate diagnosis of PTE.

Copeptin (COP) is the measurable C-terminal portion of provasopressin, which has been reported to have a prognostic value in many cardiopulmonary diseases (8). Evidence suggests that measurement of COP level may be a biomarker for PTE. To date, PTE has not been widely studied, and the prognostic value of COP has not been confirmed (2). Further studies are required to confirm the prognostic value of COP as a reliable diagnostic test in the diagnosis of PTE.

In this study, we aimed to investigate the correlation between COP and PTE and to determine the validity of COP in the diagnosis of PTE.

## MATERIALS AND METHODS

This cross-sectional study was conducted on 102 suspected PTE patients, who were referred to hospitals affiliated to Isfahan University of Medical Sciences from January 2016 to April 2017. The selected patients were admitted to the emergency department (ED) with dyspnea and were suspected of PTE. The inclusion criteria were as follows: age >18 years; dyspnea; glomerular filtration rate (GFR) >60 mL/min/1.73 m ^2^ ; and lack of hypersensitivity to dye agents. On the other hand, the exclusion criteria were as follows: use of anticoagulants; patient’s death before the diagnostic tests; and unwillingness of the patient’s physician to allow participation in the study.

We diagnosed acute PTE based on the findings of CTPA as a standard diagnostic test. Patients with a high probability of PTE (Wells score ≥6) and those with low to moderate probability of PTE (Wells score <6) and positive D-dimer were investigated by CTPA. Venous blood samples were collected for the measurement of D-dimer and COP after admission of patients to ED. D-dimer and COP were measured using the enzyme-linked immunosorbent assay (ELISA).

### Statistical analysis

The collected data were entered in SPSS version 17.00 (SPSS Inc., Chicago, IL, USA). Continuous variables are presented as mean±standard deviation, and categorical variables are presented as frequency and/or percentage. Kolmogorov-Smirnov test was also used to determine the normal distribution of continuous variables. Since the continuous variables were not normally distributed, Mann-Whitney U test was performed. Moreover, Chi-square test was performed to analyze categorical variables. The receiver operating characteristic (ROC) curve was plotted to determine the prognostic value of biomarkers. P-value less than 0.05 was considered statistically significant.

## RESULTS

This study was carried out on 102 consecutive patients with suspected PTE. As shown in Table 1, diagnosis of PTE was confirmed by CTPA in 19 (18.6%) patients. Overall, 41 (55.4%) patients had a positive D-dimer result. Chi-square test did not show any significant differences between confirmed (positive) and not-confirmed (negative) PTE cases between the two diagnostic tests (P=0.578).

**Table 1.** Comparison of suspected PTEs between the two diagnostic tests

**Diagnostic Test**	**Pulmonary thromboembolism**		**P-value**

	Positive	Negative	
CT-Angiography (n=102)	19 (18.6%)	83 (81.4%)	0.578
D-Dimer (n=47)	41 (55.4%)	6 (8.1%)	

As presented in Table 2, the serum level of COP was 485.31±428.10 pmol/L in PTE-positive patients and 426.00±489.09 pmol/L in PTE-negative patients, diagnosed by CTPA. Also, the serum level of COP was 503.23±565.71 pmol/L in PTE-positive patients and 231.17±179.27 pmol/L in PTE-negative patients, based on the D-dimer test. The diagnostic value of D-dimer and COP in identifying PTE is shown in Table 3. As shown in this table, the AUC of D-dimer was 0.581 with a standard error (SE) of 0.07 (P=0.34).

**Table 2.** Comparison of the Copeptin serum level between the two diagnostic tests

**Copeptin serum level in Diagnostic Test**	**Pulmonary thromboembolism (PTE)**		**P-value**

	Positive	Negative	
CT-Angiography Copeptin Level (pmol/L)	485.31±428.10	426.00±489.09	0.537
D-Dimer Copeptin Level (pmol/L)	503.23±565.71	231.17±179.27	0.411

**Table 3.** Comparison of validity of the two biomarkers in diagnosis of PTE

**Biomarker**	**Cut off Point**	**AUC**	**P-value**	**SE**	**Sens**	**Spec**	**PPD**	**NPD**
Copeptin (pmol/L)	>216	0.423	0.44	0.1	50%	42%	22.9%	84.2%
D-Dimer	-	0.581	0.34	0.07	100%	15.8%	20%	100%

Abbreviation: SE: Standard Error, AUC: Area under the ROC curve, Spec: specificity, Sens: sensitivity

**Figure 1.**
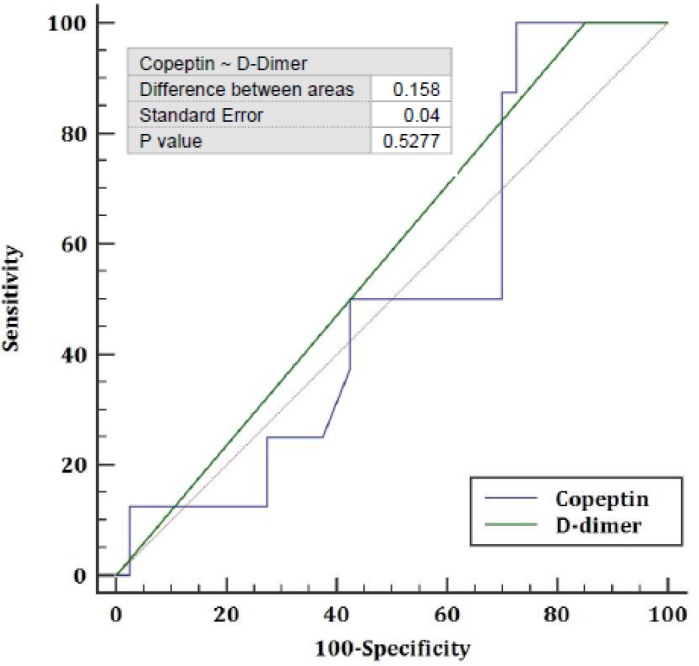
Comparison of ROC curves Copeptin and D-Dimer in diagnosis of PTE

Measurement of the validity of D-dimer test showed that it is a highly sensitive (100%), but poorly specific (15.8%) test. Estimation of the predictive value of this test indicated a PPV of 20% and NPV of 100%. Furthermore, the AUC of COP was 0.423 with SE of 0.1 (P=0.44). Evaluation of the validity of COP test showed that it is a poorly sensitive (50%) and specific (22.9%) test. Finally, comparison of the two diagnostic criteria of COP and D-dimer showed that these two criteria did not differ significantly in terms of diagnostic value (Figure 1).

## DISCUSSION

The diagnostic value of new biomarkers in the ED setting is under debate, and there are many controversies regarding the use of these biomarkers for the diagnosis of PTE. Today, there is no biomarker recommended in the guidelines for PTE (8). D-dimer has been suggested in previous protocols, but poor PPV of this biomarker has led to its limited diagnostic value (5). COP was recently introduced as a biomarker, which may have a prognostic role in ruling out or confirming the diagnosis of life-threatening PTE (9). Although CTPA can simply confirm the diagnosis of PTE, NPV of 15% has been reported for this test, challenging its validity in emergency settings (6).

The CPTA modality is a useful diagnostic test for the diagnosis of PTE, providing an excellent view of pulmonary arteries and thrombotic obstructions of arteries; nevertheless, the NPV of CTPA is low for high-probability risk of PTE (6, 10). Recent guidelines have recommended that CTPA and D-dimer tests be performed for the accurate diagnosis of PTE (1). Based on our results, D-dimer test is considerably limited in the accurate diagnosis of PTE with a specificity of 15.8%. This estimation is congruent with previous studies, which showed that negative D-dimer test results rule out PTE in patients with low to intermediate probability of PTE; however, it is not a specific test, and further examinations should be performed for individuals with high probability of PTE (5).

Unlike the present study, Kalkan et al. showed that a D-dimer level of >1041.5 ng/mL, with sensitivity and specificity of 85.1% and 60.5%, respectively, had an acceptable diagnostic value for acute pulmonary embolism (APE) (11). In another study, the critical D-dimer area was evaluated for identifying a pulmonary embolism greater than 1.2 mg/L, with a diagnostic threshold set at or above 1.2 mg/L. The sensitivity and specificity of this test were 100% and 25% in patients with low clinical probability, 100% and 33% in patients with intermediate clinical probability, and 100% and 37% in patients with high risk probability, respectively (12).

Unlike previous studies, which indicated the diagnostic value of D-dimer test in identification of PTE, in the present study, D-dimer was not successful in identifying PTE, which can be due to the fact that many patients did not have the registered results of this test, and a small sample size was recruited. In addition, previous studies have aimed to find the most accurate diagnostic cut-off point, but in this study, we relied on the positive or negative results of this test (>500 ng/mL) and did not have D-dimer levels.

On the other hand, the results of our study showed that COP, with a cut-off point of >216 pmol/L and sensitivity and specificity of 50% and 42%, respectively, did not have a suitable diagnostic value for PTE detection; nonetheless, the level of COP in patients with PTE was significantly higher than those without PTE. In addition, comparison of these two diagnostic criteria (COP and D-dimer) did not indicate any significant differences. Overall, the diagnostic role of COP is not well studied, and there are only few studies investigating the relationship between COP and PTE.

In this regard, Kalkan et al. showed that COP levels above 4.84 had 68.1% sensitivity and 83.7% specificity for predicting APE (AUC=0.836, 95% CI: 0.755–0.917; P<0.001); the negative and positive predictive values were 82.1% and 70.6%, respectively (11). In addition, they compared the two mentioned biomarkers and revealed that COP was the most specific marker of APE with specificity of 83.7% and PPV of 82.1%. Moreover, D-dimer was the most sensitive biomarker for APE, with 85.1% sensitivity and 78.8% NPV (11).

Also, Hellenkamp et al. in their study of the prognostic value of COP in pulmonary embolism found that patients with COP levels above the optimal cut-off point (24 pmol.L^−1^) had a 5.4-fold increased risk of an adverse outcome (95% CI: 1.68–17.58; P=0.005). Also, COP level equal to or greater than 24 pmol.L^−1^ stratified patients with an elevated level of high-sensitivity troponin T and N-terminal pro-brain natriuretic peptide as intermediate–low and intermediate–high risk groups, respectively (5.6% and 20.0% risk of adverse outcomes, respectively) (13).

Parlak et al. also showed that COP level in the APE group (7.58±8.61 ng/mL) was not significantly different from the control group (8.36±9.55 ng/mL). In addition, COP has been known as a prognostic marker of acute myocardial infarction. On the other hand, APE can be a serious problem, causing venous stasis, hypercoagulation, and endothelial damage. Therefore, COP may not be a suitable biomarker for pulmonary embolism, resulting from organic vascular injury. It is necessary to conduct comprehensive multi-center studies in the future in order to investigate the relationship between APE and COP level (14).

In previous studies, the prognostic and diagnostic accuracy of COP were analyzed in patients with acute coronary syndrome, heart failure, and pulmonary hypertension (15–19), and a strong relationship was reported between COP level and short- and long-term mortality in patients, who were referred to ED (20). In line with the present findings, some studies did not find any correlation between COP and PE, while unlike our study, some studies reported a significant relationship, and the acceptable diagnostic value of this marker was confirmed. The cause of discrepancy between many of these studies can be primarily the small sample size. Also, the high dispersion of COP values in this study was attributed to the small sample size, and its effect on the results was significant. One of the limitations of the present study was not having two groups of subjects with and without PTE.

In the present study, suspected PTE patients were included, and after CT angiography, they were divided into two groups with and without PTE, which led to the unequal number of patients in the two groups. Since COP has a strong correlation with the severity and long-term prognosis of left ventricular heart failure, and COP level significantly increases in patients with pulmonary hypertension and right ventricular failure (21, 18), it is recommended to conduct further studies regarding the diagnostic value of this marker in pulmonary patients with and without other comorbid diseases and compare the results with a larger sample of healthy subjects so that the findings can be reliably generalized to the general population.

## CONCLUSION

COP is a new biomarker which may have a prognostic value in the diagnosis of PTE; however, this prognostic value is not well studied. Therefore, we conducted this study to investigate the diagnostic value of COP in the diagnosis of PTE. Based on our results, there is no evidence supporting the efficacy of COP in the diagnosis of PTE, but we suggest further research to determine whether COP plays a diagnostic role in the diagnosis of PTE in emergency settings. Although COP is not a specific biomarker, the question remains as to whether it can be used as an adjunct diagnostic test in the diagnosis of PTE.
